# Amendment of the Medical Acts

**Published:** 1902-12-20

**Authors:** 


### The Hospital.

A Journal of

The Medical Sciences and Hospital Administration.

Vol. XXXIII.—No. 847.] Edited by Sir Henry Burdett, K.C.B., and Solomon C. Smith, M.D., M.R.C.P. [December 20, 1902.

### Amendment of the Medical Acts.

On the last day of its recent session, the General Medical Council received from its "Financial Relations Committee" the draft of a suggested Bill for the Amendment of the Medical Acts, a draft which had been prepared by the Committee in question, and modified in certain respects under the advice of Mr. Muir Mackenzie, the legal adviser of the Council. The subject is one which has been discussed by the Council on previous occasions, having been forced upon its notice by two chief reasons, one being the approaching exhaustion of its corporate funds, and the other being the unsatisfactory relations, as regards finance, which exist between the Council as a whole and the Scottish and Irish Branch Councils. The Bill, as presented to the Council, was ordered to be printed on its minutes "as a record," and was then referred back to the Financial Relations Committee, together with the report of that Committee on which it was founded, in order that additional suggestions with regard to "economies in expenditure" might be received and considered before the next meeting of the Council in May. A motion was also carried saying that, "in view of the extreme difficulty *in* (*sic*) obtaining a hearing for Bills introduced by private members, an active effort be made, by deputation or otherwise, to secure the interest of the Government in the Bills promoted by the Council." It may be inferred not only that the Council means business, but also that some of the more sanguine among its members entertain a hope that their amateur efforts at legislation may receive official sanction and support.

In view of this hope, for which there may, for anything we know to the contrary, exist some shadow of foundation, it becomes important to look somewhat closely at the legislative proposals of the Council, or rather of its committee, and to see what are the amendments in the existing law which they desire to see adopted by the Legislature. The original Medical Act is now forty-four years old, and the chief amending Act is sixteen years old, so that considerable experience of the working of both has been obtained; and as far as the profession at large is concerned we do not think it can be said that this working has been entirely satisfactory to all concerned. We have even heard criticisms which might be described as harsh or unfriendly. It is therefore highly interesting to discover that, in the opinion of a very strong and representative committee of the Council, these criticisms are captious or unfounded. Apart from a rearrangement of the business respectively transacted by the Council and the branches, a rearrangement which may be described as a mere matter of official convenience, the only change in the law recommended by the committee is that the Council should be empowered to raise the registration fee of practitioners from £5 to some amount not exceeding £10, and to charge a fee not exceeding £1 for registration in the Student's Register.

It is, of course, possible that the *possidentes*, the gentlemen whose names are already on the Medical Register, may view with comparative indifference a proposal to increase the cost of qualification to those who come after them; and it is certain that the proposal to charge for admission to the Student's Register would remain a dead letter in practice, even if it were sanctioned by law. As we mentioned last week, the Council has no power to enforce the registration of students, and some of the examining bodies have already given practical effect to their disapproval of the conditions of such registration, as laid down by the Council, by ceasing to require it from those who seek their diplomas. Others, in the event of the proposed charge being legalised, would be compelled to follow suit; and the general result would be that no student would consent to pay £1 from which he would derive no possible advantage. The registration of students would simply cease. When we come to the more important proposal, to levy an increased tax upon all future medical practitioners for the better support of the Council, it seems obvious that such a suggestion, if brought forward in a Bill promoted by a private member, would merely furnish a fresh illustration of the "difficulty" to which reference is made in the resolution quoted above. It is perhaps equally obvious that it could not be adopted by the Government without full consideration of many other issues, nor without the introduction of provisions of other kinds. The Council was instituted for the purpose of securing a practical similarity or equality of medical education and examination, not only as between the three divisions of the kingdom, but also as between the several qualifying bodies of each division; and this requirement, for want of sufficient authority, it has signally failed to fulfil. It is an open question whether the necessary authority could be usefully bestowed upon, or would probably be wisely exerted by, any central executive body, and whether the requirements of medical education, like many requirements of other kinds, would not be most completely fulfilled by the natural reactions of demand and supply, and by the natural competition between universities and colleges, each of them offering an article the value and repute of which it would be to their interest to maintain. If the opposite opinion be upheld, and if the existence of a central Council be still deemed necessary or expedient, it is manifest that it cannot be suffered to remain, in the future, as powerless as it has been in the past. As far as regards the discharge of the functions imposed upon it by the Legislature, it is now in the position of the Irishman's sedan in which but for the name of the thing he might as well have walked; or in that of a scarecrow which the birds have found out. Its authority has been openly set at defiance by some of its constituent bodies, and can only be maintained in appearance by the judicious avoidance of conflict. If such a body is to receive more money, it must at the same time be rendered capable of doing more work.

